# Ultrasonic Guided Waves for Liquid Water Localization in Fuel Cells: An Ex Situ Proof of Principle

**DOI:** 10.3390/s22218296

**Published:** 2022-10-29

**Authors:** Jakob Sablowski, Ziwen Zhao, Christian Kupsch

**Affiliations:** Measurement, Sensor and Embedded Systems Laboratory, Institute of Electrical Engineering, TU Bergakademie Freiberg, Winklerstrasse 5, 09599 Freiberg, Germany

**Keywords:** ultrasonic guided waves, signal processing, deposit localization, water management, fuel cells

## Abstract

Water management is a key issue in the design and operation of proton exchange membrane fuel cells (PEMFCs). For an efficient and stable operation, the accumulation of liquid water inside the flow channels has to be prevented. Existing measurement methods for localizing water are limited in terms of the integration and application of measurements in operating PEMFC stacks. In this study, we present a measurement method for the localization of liquid water based on ultrasonic guided waves. Using a sparse sensing array of four piezoelectric wafer active sensors (PWAS), the measurement requires only minor changes in the PEMFC cell design. The measurement method is demonstrated with ex situ measurements for water drop localization on a single bipolar plate. The wave propagation of the guided waves and their interaction with water drops on different positions of the bipolar plate are investigated. The complex geometry of the bipolar plate leads to complex guided wave responses. Thus, physical modeling of the wave propagation and tomographic methods are not suitable for the localization of the water drops. Using machine learning methods, it is demonstrated that the position of a water drop can be obtained from the guided wave responses despite the complex geometry of the bipolar plate. Our results show standard deviations of 4.2 mm and 3.3 mm in the *x* and *y* coordinates, respectively. The measurement method shows high potential for in situ measurements in PEMFC stacks as well as for other applications that require deposit localization on geometrically complex waveguides.

## 1. Introduction

### 1.1. Water Management in Fuel Cells

In order to reduce the environmental impact of fossil fuels, regenerative energy sources, i.e., wind and solar energy will be used predominantly in the future. To overcome their volatility, scalable and efficient energy storage and conversion systems are required. Fuel cells offer several advantages, i.e., high conversion efficiency, harmless exhaustion products, and high availability of the required materials [1], and have thereby become one of the most promising technologies to support the transition from fossil fuels to regenerative energy sources [2].

Proton exchange membrane fuel cells (PEMFCs) are operated by supplying hydrogen on the anode and oxygen on the cathode side. The hydrogen penetrates the gas diffusion layer and reacts with the electrolyte, emitting electrons for the outer electrical circuit and protons, which travel to the cathode side through the proton exchange membrane that separates the cathode and anode. At the cathode side, protons and electrons recombine with oxygen to produce water. Due to the low operating temperatures between 60 °C and 80 °C [3], the water can be liquid.

It has been shown that water management is important for the operation and optimization of PEMFCs [4]. Furthermore, water management has a great influence on the long-term performance and durability of PEMFCs [5]. An optimal working point for the humidity in PEMFCs has to be found based on the following criteria: the water that has been generated in the chemical reaction has to be transported out of the cell to prevent liquid water from blocking the flow channels and to ensure an even distribution of the reactants. At the same time, the fuel cell has to be prevented from dehydration since the proton exchange membrane requires a certain humidity. Numerous water management methods and strategies for PEMFCs have been developed [1,6,7], which are based on improving the PEMFC design or material, e.g., the flow field design, design of gas diffusion layer, or respective materials. However, to understand the processes relevant to water formation and transport, methods for visualizing water in the fuel cell are required. In addition, Wang et al. concluded that monitoring the water inside the PEMFC is the most reliable way to determine issues with water management [1], e.g., water flooding the flow channels.

For the visualization and investigation of water in PEMFCs, various methods based on optical visualization, imaging using ionizing radiation, and magnetic resonance imaging have been applied [8]. The optical visualization of water uses laser- and camera-based methods. This requires optical access, which is typically not given in PEMFCs. Hence, simplified models with optical windows are used and the operational conditions are different from real PEMFCs. Rahimi et al. developed a transparent PEMFC stack to investigate water flow visually under different operational conditions [9]. However, due to the necessary changes in the cell design, the boundary conditions, e.g., thermal conductivity and wettability, change. Hence, the processes in real PEMFCs can only be approximated. Visualization techniques based on ionizing radiation, e.g., X-ray and neutron imaging, allow the imaging of the water distribution in PEMFCs in the channels and the gas diffusion layer [10,11,12]. However, the applicability of these techniques is limited since appropriate radiation sources are required, which are not commonly available for neutron imaging, or only single cells or smaller sections of single PEMFC cells can be investigated. Hence, water imaging based on ionizing radiation is not suitable for detailed parameter studies on water management or in situ measurements. Magnetic resonance imaging has been applied for the imaging of water in flow channels and the gas diffusion layer in situ [13,14]. However, the temporal resolution was limited, e.g., to 50 s [13] and dynamic processes were hardly observed. Furthermore, changes to the investigated setup of the PEMFC were required. This included the size to fit the cell into the magnet’s core and the changes to the nonmagnetic materials for the bipolar plate and current collector. In conclusion, detailed analysis of water is possible with state-of-the-art techniques but requires great efforts or modifications of the cell design so the results only have limited use for water management in real PEMFC stacks. Therefore, we introduce a new method for the detection and localization of water in PEMFCs based on guided waves, which is demonstrated in ex situ experiments on a single bipolar plate of a PEMFC.

### 1.2. Measurements Based on Guided Waves

Measurements based on ultrasonic guided waves are common in the fields of nondestructive evaluation and structural health monitoring (SHM) [15,16,17,18,19]. Ultrasonic guided waves are mechanical waves in the kHz to the MHz range that propagate through media and are delimited to another material, e.g., plate-like structures. Various kinds of transducers can be used for the generation of guided waves, as well as for sensing the response after the waves have propagated through the structure under investigation. The propagation in these structures produces dispersive modes of propagation. The measurement effect in SHM is based on the interaction of the guided waves with a defect of the structure, e.g., from an impact. This interaction may lead to reflections, frequency-dependent attenuation, or mode conversion. By analyzing the guided wave responses received from the sensors, it is possible to detect defects in plate-like structures such as metal plates, e.g., on aircraft wings [20].

A wide range of signal processing techniques, such as wavelet transform and mode identification, have been applied for this purpose. Many of these techniques rely on comparing the guided wave responses from the monitored structure with those of the undamaged structure to detect differences. In addition to the detection of defects, guided waves have also been used for the localization and imaging of defects. Most commonly, tomographic approaches are used for imaging based on guided waves [21,22,23].

Apart from defects in the structure itself, guided waves are also sensitive to changes in the adjacent media, e.g., due to deposits on the structure. This effect has been used for the monitoring of soft depositions in liquid-filled tubes [24], ice detection on windmill wings [25,26], and for monitoring the wetting on simple plate-like structures [27]. Guided wave tomography has been applied to localize ice deposits on simple plate structures [28]

Typically, measurements based on guided waves are investigated on structures with simple geometries and with or without a low number of discontinuities, e.g., plate-like structures or tubes with constant thicknesses [16]. This is not the case for the bipolar plates investigated in this study. Bipolar plates in fuel cells are used to distribute the reactants through flow channels across the entire active surface of the cell. When metallic bipolar plates are used, the flow channels are created by deep drawing from a metal sheet, which introduces mechanical stress and bends. Hence, the created structure consists of a high number of discontinuities and is more complex compared to simple plate-like structures or tubes. Efforts have been made to understand and model the propagation of guided waves in plates with a single bend [29,30]. However, the understanding of guided wave propagation in geometrically complex structures with many bends, such as a bipolar plate with a flow field that consists of several flow channels, is still very limited. It can be assumed that the guided wave responses of such a structure are much more complex and it is unknown how they change due to deposits such as water drops. Therefore, methods that have been applied successfully to detect deposits on simple plate-like structures, e.g., mode identification and time-of-flight measurements [24], cannot be easily adapted to this problem. With regard to localization, tomography-based imaging methods might fail as well since the signal paths between the transmitting and receiving transducers depend on the unknown guided wave propagation within the flow field.

Data-driven methods for signal processing are an option for dealing with the inherent complexity associated with guided wave measurements. These methods rely on machine learning (ML) and data from previous measurements rather than physical models. Various ML methods have been applied to guided wave measurements in the field of SHM, often with the aim of damage detection and classification [31,32,33]. Hesser et al. have applied ML algorithms to localize impact positions on an aluminum plate based on guided wave responses [34]. Supervised machine learning has also been applied for the classification of deposits on wind turbine blades using guided waves [26,35]. To the best of our knowledge, the localization of deposits on complex structures such as the bipolar plate has not been demonstrated so far.

### 1.3. Aim and Outline of This Article

In this study, we propose a new method for the localization of liquid water in PEMFCs using guided waves. The measurement method is investigated in ex situ experiments on a single bipolar plate. In addition to its application in PEMFCs, the broader research question of this article is whether deposit localization based on guided waves can be successfully applied to complex structures such as the bipolar plate. First, the experimental setup of a sparse sensor array on a single bipolar plate is introduced. Based on the experimental results, the guided wave propagation through the bipolar plate is investigated. It is then shown how single water drops on the flow field affect the signal properties of the guided wave responses based on the location of the drops. Subsequently, a data-driven approach to localization is presented. Finally, the uncertainty of water localization is evaluated.

## 2. Materials and Methods

### 2.1. Experimental Setup

**Figure 1:**
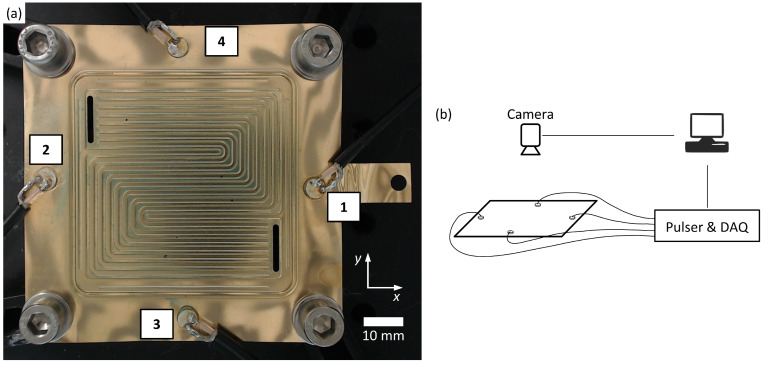
(**a**) Bipolar plate with four PWAS connected to it. The flow field in the center consists of eight serpentine flow channels, which connect the gas inlet and outlet. (**b**) Scheme of the experimental setup showing the camera, pulser, and data acquisition system (DAQ) connected to a PC.

To investigate the effect of water drops on guided wave propagation, a single PEMFC bipolar plate was used as a sample. The bipolar plate consisted of stainless steel (Material No.: 1.4404, AISI: 316L) with a gold coating of 1 μm, a sheet thickness of 100 μm, and outer dimensions of 10 cm by 8 cm. The bipolar plate had a serpentine flow field with eight flow channels that connect the gas inlet and outlet (flow field design: *Hydrogen and Fuel Cell Center (ZBT GmbH)*, Duisburg, Germany). The depth of the flow channels was 0.32 mm. Four piezoelectric wafer active sensors (PWAS) were connected to a bipolar plate outside of the flow field with epoxy glue. The sample with the connected PWAS is shown in Figure 1a.

To investigate the localization of water inside the flow field, water drops were placed in different positions inside the channels. For each measurement, a single drop of water with a volume of approximately 5 μl was placed on the flow field. All measurements were conducted in a temperature-controlled laboratory environment at 20 °C. The measurement system was used to excite guided waves in the bipolar plate. One of the PWAS was excited while the received signals at all four PWAS were recorded. To reduce signal noise, 500 recorded signals were averaged. This procedure was repeated with each of the four PWAS being used as the transmitter, resulting in a measurement that consisted of 16 averaged signals (including the echo signals of each PWAS). In addition to the guided wave measurements, the position and size of the water drops were obtained. For this purpose, a camera was mounted to a fixed position above the bipolar plate and an image of the flow field was captured and saved for each measurement. To guarantee the same position of the flow field in each image, all images were adjusted via template matching. The images were cropped to 800 × 800 pixels and the position of the water drop was extracted from each image. The image analysis was automated using the OpenCV library [36]. A total of 296 measurements were conducted, each with a single drop placed on a different position in the flow field. Additionally, seven measurements were conducted without any water on the flow field.

### 2.2. Ultrasound System

The ultrasound system drove four PWAS (disc c255 o5 t0,5 wAg, *PI Ceramic*, Lederhose, Germany) made from the piezoelectric material PIC 255 (*PI Ceramic*, Lederhose, Germany). These PWAS had a thickness of 0.5 mm and a diameter of 5 mm, with a thickness resonance frequency of 4 MHz and a radial resonance frequency of 0.5 MHz. The PWAS were driven by a 16-channel pulser receiver unit that is described in detail elsewhere [37]. To transmit ultrasound signals, the PWAS were excited with a 200 V pulse. For this purpose, the pulser generated a single-sided negative voltage pulse with a rise time of approximately 4 ns. Simultaneously, all PWAS were used as sensors to receive the guided wave responses. The pulse repetition rate was set to 500 Hz. A 54 dB gain was applied to the received signals. Data acquisition was realized with two GaGe Octopus digitizer boards (*Vitrek LLC*, Lockport, IL, USA) with a sample rate of 125 MHz and a resolution of 14 bits. The pulser and the data acquisition system (DAQ) were connected to a PC that was used to control the devices and for data storage. A scheme of the measurement system is shown in Figure 1b.

## 3. Signal Analysis and Evaluation

### 3.1. Propagation of Guided Waves in a PEMFC Bipolar Plate

**Figure 2:**
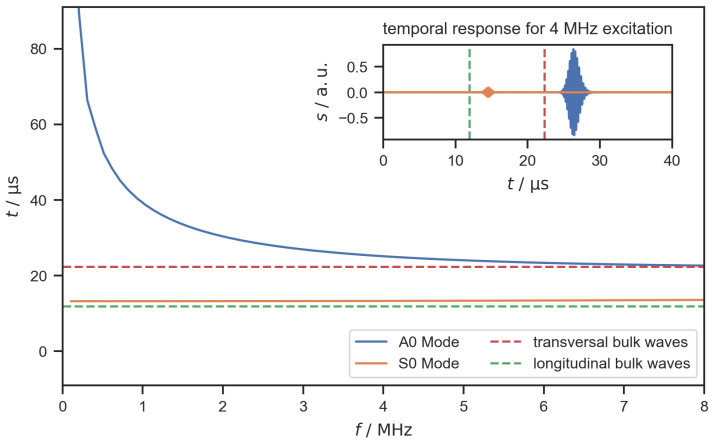
Propagation times of $A_{0}$ and $S_{0}$ modes and bulk wave for a distance of 68 mm on a flat stainless steel plate (thickness 0.1 mm) calculated with the Dispersion Calculator [38]. The inset shows the calculated out-of-plane amplitude of the temporal guided wave responses for a 4 MHz sinusoidal excitation signal (ten cycles, Gaussian window).

The PWAS were used to excite guided waves in the bipolar plate. To lower the complexity of the guided wave responses, the thickness resonance frequency of the PWAS was chosen so that only the zero-order modes *A*_0_ and *S*_0_ were excited. On a flat, isotropic, and sufficiently large plate, it could be expected to observe Lamb-type guided wave responses with two distinct peaks for the two zero-order modes, which propagate at different velocities through the plate. To demonstrate this ideal case, the dispersion curves were calculated assuming a flat plate with a thickness of 100 μm consisting of stainless steel (Material No.: 1.4301, AISI: 304). The Dispersion Calculator software was used to calculate the dispersion curves [38]. Figure 2 shows the propagation times of the *A*_0_ and *S*_0_ modes and for the bulk waves for a distance of 68 mm, which corresponds to the center-to-center distance between PWAS 1 and 2. In the calculated temporal response, the two modes can be clearly distinguished from one another under the assumption of a flat, isotropic plate.

**Figure 3:**
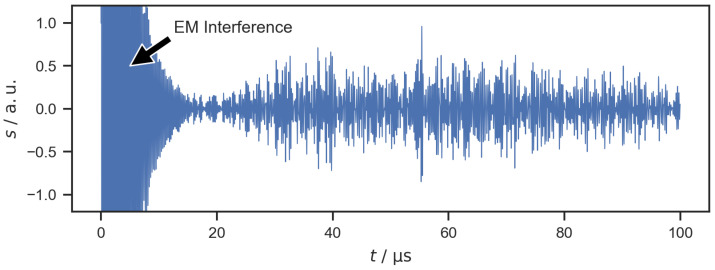
Filtered signal transmitted from PWAS 1 and received at PWAS 2. Electromagnetic (EM) interference occurs at the beginning of the signal.

In the experiment, the boundary conditions for wave propagation significantly differed from the theoretical scenario of a flat, isotropic, and infinite plate. The geometry of a bipolar plate is more complex, featuring the serpentine flow field with its two holes for the gas inlet and outlet. The edges of the bipolar plate are close to the positions of the PWAS and, therefore, the bipolar plate cannot be approximated by an infinite plate. Additionally, the deep drawing of the flow field might have introduced anisotropy within the material. Compared to a flat, infinite, and isotropic plate, these properties introduce additional phenomena to wave propagation such as reflection and mode conversion. It has been shown that geometric features can lead to a concentration of wave energy and act as local waveguides within a plate. This phenomenon of feature-guided waves has been investigated mostly in the context of non-destructive testing and for plates with simple geometric features such as bends [29,30], stiffeners [39,40], and welded joints [41,42,43]. It can be assumed that similar effects also occur alongside the channels within the flow field. These boundary conditions for wave propagation differ from Lamb wave theory and result in more complex guided wave responses. Figure 3 shows the amplitude *s* of a signal that was transmitted from PWAS 1 and received at PWAS 2. The signal was recorded without any water present on the flow field and has been forward-backward filtered with a fourth-order Butterworth bandpass filter (lower and upper −3 dB cutoff frequencies: 3 MHz and 7 MHz). At the beginning of the signal, the effect of electromagnetic interference within the pulser receiver unit can be seen. From about 15 μs onward, the signal shows the effect of the guided waves arriving at PWAS 2. In contrast to the theoretical signal shown in Figure 2, the measured signal does not show two clear peaks for the two Lamb wave modes. Instead, the measured signal is longer and looks much more complex. Reflections on the edge of the bipolar plate and the complex geometry of the flow field result in a guided wave response that consists of many superimposed oscillations. The measured signal does not allow for a clear identification of the different Lamb wave modes. This shows that a signal analysis based on modeling the wave propagation in a bipolar plate is not suitable for the localization of water drops on a flow field.

### 3.2. Influence of Water Drops on the Guided Wave Response

**Figure 4:**
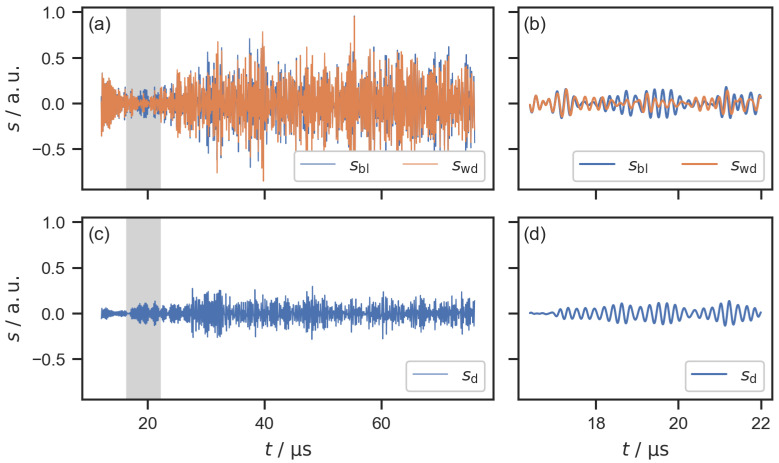
(**a**) Filtered signals transmitted from PWAS 1 and received at PWAS 2. The baseline signal $s_{\mathrm{bl}}$ was recorded without a water drop on the flow field and the measurement signal $s_{\mathrm{wd}}$ was recorded with a single water drop on the flow field. (**c**) Difference signal $s_{d}$. (**b**,**d**) Detailed views of the highlighted signal parts.

Guided waves in plate-like structures are not only sensitive to structural defects within the plate but also changes at the surface interface. Water contact with the surface can lead to attenuation, changes in time-of-flight, and mode conversion of the guided waves [44,45]. Sessile water drops on a plate lead to the scattering of guided waves [46]. The sensitivity of guided waves to water on the surface of the plate has been investigated to quantify surface wetting [27]. To investigate the influence of the water drops experimentally, signals from one measurement with the dry bipolar plate were used as baselines. Figure 4 shows a baseline signal $s_{\mathrm{bl}}$ and a second signal $s_{\mathrm{wd}}$ that was recorded with a single water drop on the flow field, as well as the difference signal $s_{d}$ obtained by baseline subtraction according to the following equation,

(1) $$s_{d}\left(n\right)=s_{\mathrm{wd}}\left(n\right)-s_{\mathrm{bl}}\left(n\right),$$

where *n* is the sample index. Changes in the amplitude and phase can be seen between $s_{\mathrm{bl}}$ and $s_{\mathrm{wd}}$. It is clear that the water drop on the flow field has an influence on the guided wave response that is represented by the difference signal $s_{d}$.

**Figure 5:**
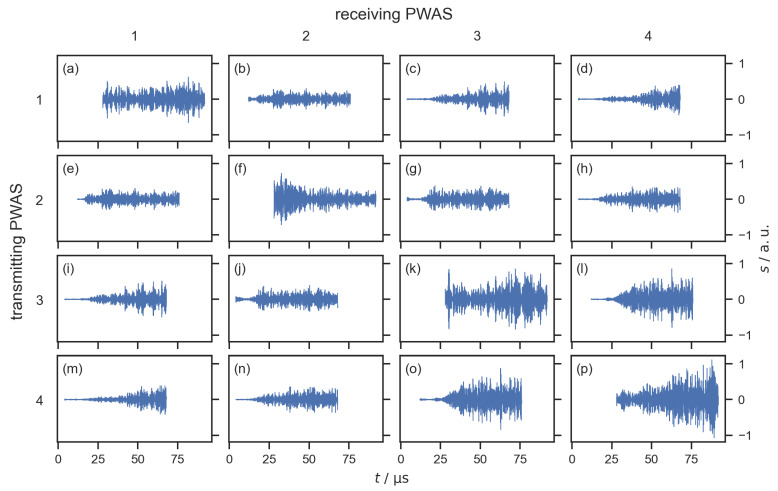
Difference signals within the ROI for all signal paths. (**a**,**f**,**k**,**p**) Echo signals that are transmitted and received by the same PWAS. (**b**,**e**,**l**,**o**) Signal paths across the flow field. The remaining difference signals are for diagonal signal paths. The difference signal shown in (**b**) is the same signal that is shown in Figure 4c. For the water detection and localization, signals shown in (**e**,**i**,**j**,**m**–**o**) are disregarded since they are almost identical to signals shown in (**b**–**d**,**g**,**h**,**l**), respectively.

Figure 5 shows the filtered difference signals for all 16 signal paths for one measurement with a water drop on the flow field. The difference signal for the signal path from PWAS 1 (transmitting PWAS) to PWAS 2 (receiving PWAS), which is shown in Figure 4c, can be seen in Figure 5b. The other difference signals shown in Figure 5 were obtained accordingly for each combination of transmitting and receiving PWAS. A region of interest (ROI) with a length of 64.0 μs was chosen for the subsequent signal processing. This corresponds to a length of 8000 samples. The positions of the ROI depend on the signal paths, starting at 12.0 μs for the signal paths across the flow field (between PWAS 1 and 2, as well as between PWAS 3 and 4, Figure 5b,e,i,o), 28.0 μs for the echo signals (Figure 5a,f,k,p), and 4.0 μs for the diagonal signal paths. The ROIs were chosen with respect to the different lengths of the signal paths to omit the electromagnetic interference at the beginning of each signal and to avoid regions with clipping in the echo signals. As expected, the signals for each signal path were independent of the direction of the signal, e.g., the signal transmitted from PWAS 1 to PWAS 2 was almost identical to the signal transmitted from PWAS 2 to PWAS 1. This resulted in almost identical difference signals for both directions of each signal path, e.g., the difference signals shown in Figure 5b and Figure 5e. Therefore, only one direction of each signal path was used for the localization, resulting in 10 difference signals for each measurement.

To quantify the influence of the water drop on the guided wave response, the sum of squares of the difference signal $s_{d}$ was obtained. This sum corresponds to the signal energy of the difference signal $E_{d}$ and was calculated according to the following equation:

(2) $$E_{d}=\sum_{n=1}^{N}s_{d}{\left(n\right)}^{2},$$

where *n* is the sample index, $s_{d}\left(n\right)$ is the difference signal, and *N* is the number of samples within the ROI. $E_{d}$ provides a measure for the change between the measurement signal and the baseline signal, both in the phase and amplitude, and does not require any additional knowledge about wave propagation. This approach is known from other measuring methods such as Lamb wave tomography [23]. The signal energy of the difference signals is raised if water is present on the flow field and a higher value indicates a stronger influence of the water. For example, the signal energy $E_{d}$ of the difference signal shown in Figure 4c is 7.3 times higher than the mean value of $E_{d}$ for the six measurements without water. This shows how the signal energy of the difference signal allowed for the detection of liquid water on the flow field. This measurement method is based on ultrasonic guided waves and can be applied to complex geometrical structures such as the bipolar plate in the experiments.

**Figure 6:**
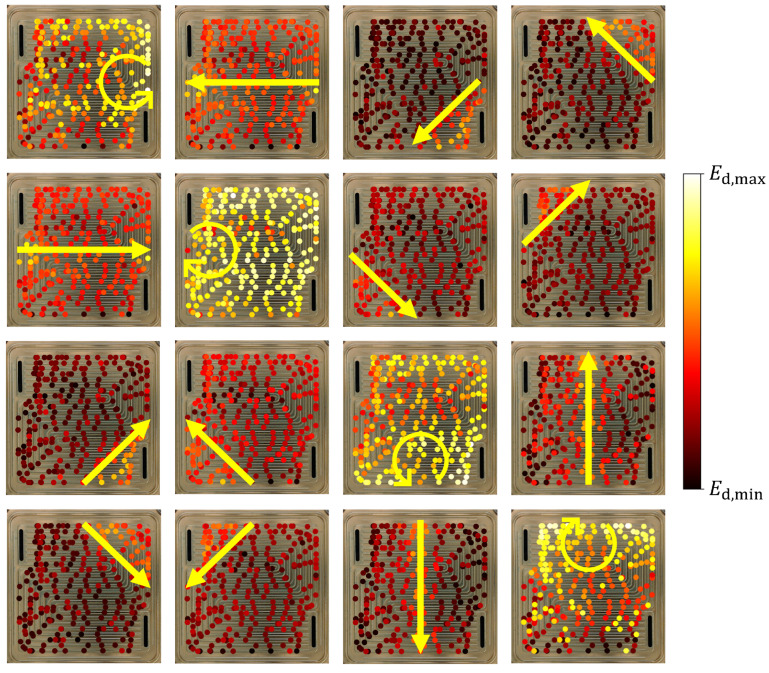
Change in the signal energy of the difference signal $E_{d}$ depending on the position of the drop. The arrows indicate the signal path from the transmitter to the receiver. The colors indicate the value of $E_{d}$ relative to the minimum and maximum values for each signal path.

The signal energy of the difference signal depends on the position of the water drop relative to the signal paths and the geometry of the flow field. This can be shown by associating the positions of the water drops, which were obtained via image analysis, with the signal energy of the difference signal. In Figure 6, all measurements were superpositioned in one image for each signal path to show this effect. The relative values of $E_{d}$ are color-coded to show which drop positions had a high influence on the guided wave response for each signal path. It can be seen that the drops that were positioned close to the transmitter or receiver or in the proximity of the signal path between the two caused larger changes in the guided wave response resulting in a larger signal energy of the difference signal compared to drops that were positioned elsewhere. This was expected, since every reflection of the guided waves, e.g., at the edges of the bipolar plate, involves attenuation. Therefore, waves that travel on a direct path from the transmitter to the receiver can be expected to influence the received signals more than waves that are reflected multiple times. However, due to the complex geometry of the flow field, the path from the transmitter to the receiver that involves the least amount of attenuation does not necessarily correspond to a linear connection between the transmitter and the receiver. As an example, in the first image in Figure 6, the echo signal from PWAS 1 is shown. The image shows that the us signals were more sensitive toward drops in the upper area of the flow field (near PWAS 4) compared to some drops in the center of the flow field. We suspect that this effect was a result of the flow channels acting as local waveguides. Note that the values of $E_{d}$ in Figure 6 were calculated for the whole 64.0 μs ROI of the difference signals. Thus, no time-dependent information from the signal is included.

**Figure 7:**
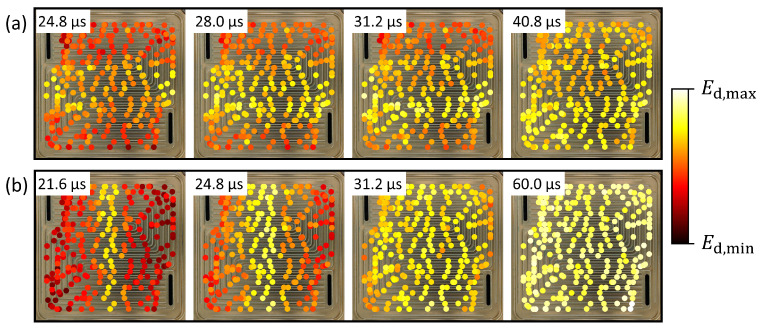
Change in the signal energy of the difference signal $E_{d}$ over time. $E_{d}$ is shown for the windows of the difference signal. Each window is 3.2 μs long, the timestamps in the figure mark the beginning of each window. (**a**) Signal transmitted from PWAS 2 to PWAS 1, (**b**) signal transmitted from PWAS 4 to PWAS 3. The colors indicate the value of $E_{d}$ relative to the minimum and maximum values for each signal path.

In order to investigate the wave propagation over time, the difference signal was divided into 20 windows before calculating the signal energy for each window. In Figure 7, some of the windows are shown for the two signal paths across the flow field. Again, the effect of the flow field geometry on the guided wave propagation can be observed: for the horizontal signal path across the flow fields shown in Figure 7a, some drops in the center of the flow field showed a smaller effect on the signal energy compared to other drops. In the case of the vertical signal path, which lies perpendicular to all channels alongside the direct path between PWAS 4 and 3, this effect was not observed. Drops positioned far away from the direct signal path, e.g., in the corners of the flow field, showed small relative values of $E_{d}$ in the first time windows. In later time windows, a more uniform distribution was observed and the relative values for $E_{d}$ were high for all drop positions.

Since the signal energy of the difference signal clearly depends on the position of the drop in relation to the signal paths and the geometry of the flow field, it can be used to localize the drops. However, ray tracing models do not sufficiently capture the influence of the flow field geometry. Therefore, tomographic techniques are not suitable for localization. Instead, statistical models based on measurement data were investigated.

## 4. Localization of Water Drops

### 4.1. Model Development and Training

To demonstrate the localization of water drops based on the guided wave responses, multiple linear regression modeling was applied to estimate the center positions of the drops. As discussed in the previous section, the signal energy of the filtered difference signals $E_{d}$ contains information on the position of the water drops. This signal feature was therefore chosen as the model input. The linear regression model is given by

(3) $$\hat{x}=f\left(E_{d,1},...,E_{d,k}\right)=\beta_{x,0}+\beta_{x,1}E_{d,1}+...+\beta_{x,k}E_{d,k}=\beta_{x}E_{d}^{\prime },$$

where $\hat{x}$ is the predicted value of the *x* coordinate, $E_{d}^{\prime }=\left(1,E_{d,1},...,E_{d,k}\right)$ represents the *k* model inputs, and $\beta_{x}=\left(\beta_{x,0},...,\beta_{x,k}\right)$ represents the k+1 model coefficients to obtain the *x* coordinate of the drop. Two models were applied to independently predict the *x* and *y* coordinates of the drop positions on the flow field. The two sets of model coefficients $\beta_{x}$ and $\beta_{y}$ were found via the ordinary least squares method using the training data $E_{d}=\left(E_{d,1}^{\prime },...,E_{d,j}^{\prime }\right)$ from the same *j* measurements. The optimization problem to train the *x* coordinates model is given by

(4) $$\beta_{x}=\underset{\beta }{arg min}{∥\beta E_{d}-x∥}_{2}^{2},$$

where $x=(x_{1},...,x_{j})$ are the true *x* coordinates of the drop positions that were obtained from the photographs via image analysis. The training of the *y* coordinate model was conducted using the true *y* coordinates $y=(y_{1},...,y_{j})$ from the same dataset. The model training was conducted using the Python scikit-learn library [47]. The 296 measurements with single drops on the flow field were randomly split into training and test datasets containing 237 and 59 measurements, respectively.

To obtain the model inputs $E_{d}^{\prime }$ from the raw ultrasound signals, the following signal preprocessing was applied:

1. Baseline subtraction;
2. Disregarding reversed signal paths because of the high similarity of the signals due to time-reversal invariance. Only one direction for each signal path was used. This leaves 10 out of the 16 signals for each measurement;
3. Slicing the signals to ROIs with a length of 64.0 μs, as discussed in Section 3.2 and shown in Figure 5;
4. Butterworth bandpass filter with lower and upper cutoff frequencies of 3 MHz and 7 MHz, respectively;
5. Windowing of each signal in 250 windows, resulting in a length of 0.256 μs for each window;
6. Calculating the signal energy of the difference signal $E_{d}$ for each window of each of the 10 relevant signal paths.

**Figure 8:**
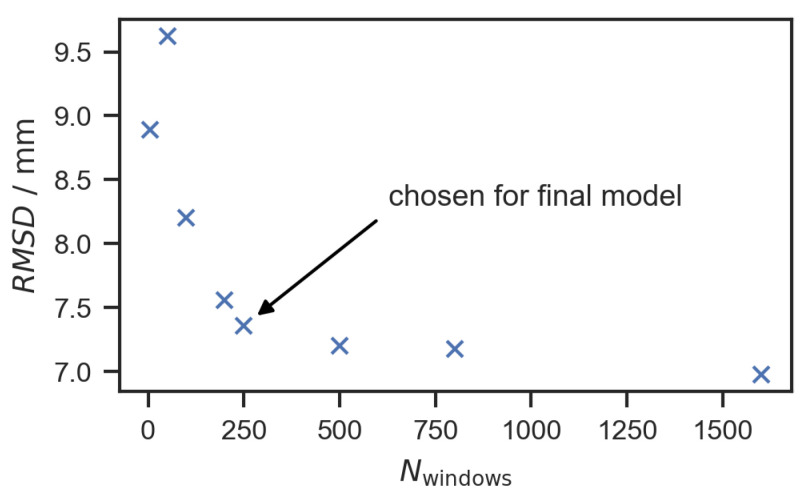
Results of the cross-validation for different numbers of time windows.

This signal preprocessing resulted in *k* = 2500 model inputs for each measurement. The signal preprocessing applied to obtain the model inputs was identical to that for the signals shown in Figure 6 and Figure 7, except for the number of windows, which was increased to 250. The parameters for signal preprocessing were found via fivefold cross-validation of the ML model using only the training dataset. The root mean square deviation was used as a measure to choose the suitable parameters for the signal preprocessing:

(5) $$RMSD=\sqrt{\frac{1}{5}\sum_{i=1}^{5}MSD_{i}},$$

where MSD is the mean squared deviation for the results of the *i*-th cross-validation given by

(6) $$MSD_{i}=\frac{1}{M_{i}}\sum_{m=1}^{M_{i}}\left({\left(x_{m}-{\hat{x}}_{m}\right)}^{2}+{\left(y_{m}-{\hat{y}}_{m}\right)}^{2}\right),$$

where $M_{i}$ is the number of measurements used for the *i*-th cross-validation. It was found that using all 16 signal paths (including the reversed signal paths) did not improve the model. By increasing the number of windows for each signal, the model could be slightly improved. However, the improvement was only small for the more than 250 windows, as shown in Figure 8. It seems that the period of 0.25 μs (for the 4 MHz excitation of the PWAS) is a good indication to choose the optimal length of the time windows, which was 0.256 μs in the case of the 250 windows.

### 4.2. Characterization of Localization Properties

**Figure 9:**
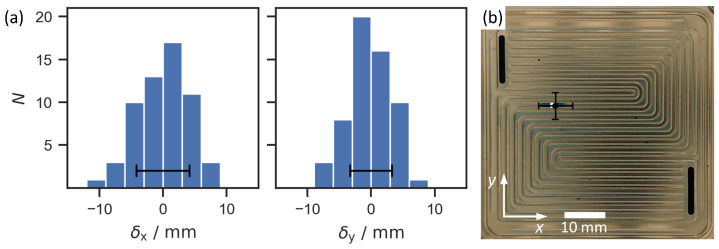
(**a**) Histograms showing the frequency distribution of the differences $\delta_{x}$ and $\delta_{y}$ between the estimated and true *x* and *y* coordinates for the test cases. (**b**) Flow field with the true (white square) and predicted (black circle) positions of the water drop for one measurement. The error bars mark double the empirical standard deviation in all images.

The two regression models for the *x* and *y* coordinates were applied to estimate the drop positions for the test dataset based on the guided wave responses. One of the measurements was disregarded as an outlier since the estimated drop position was outside of the flow field. This left 58 measurements for the characterization of the localization properties. Figure 9a shows the distributions of the deviations of the estimated drop positions from the true positions for the *x* and *y* coordinates. The coefficient of determination $R^{2}$ was 0.92. $R^{2}$ was calculated from the residual sum of squares $SS_{\mathrm{res}}$ and the total sum of squares $SS_{\mathrm{tot}}$ by

(7) $$R^{2}=1-\frac{SS_{\mathrm{res}}}{SS_{\mathrm{tot}}}.$$

$R^{2}$ is therefore a measure of the global fit of the model. A value of $R^{2}=1$ would indicate that the fitted model explained the variability in the dataset, and in this case, there would be no deviation between the estimated and the true positions of the drops. In the experiment, the estimated positions of the drops deviated from the true positions with an empirical standard deviation of 4.2 mm and 3.3 mm in the *x* and *y* directions, respectively. In Figure 9b, it can be seen that these values correspond roughly to half the length of a water drop. Since only the center position of the drop was used to train the regression model, no information on the orientation of the aspherical, elongated drops inside the channels was considered. However, the interaction between the drop and the guided waves in the bipolar plate occurred over the whole contact area of the drop including both ends of the elongated drop. Therefore, it could be expected that the measurement uncertainty would at least be in the range of the drop length. Considering this effect, as well as the use of a simple linear regression model to estimate the drop positions, it can be assumed that the accuracy of the localization can be further improved if necessary. This could be achieved by including more information such as the contact area shape of the drop to train more advanced ML models.

The number of PWAS also has to be considered with regard to the accuracy of the localization. A low number of PWAS is beneficial for integration in a fuel cell, whereas using more PWAS might improve the accuracy of the localization. In the experiments, a sparse sensor array of four PWAS with resonance frequencies of 4 MHz was used to detect drops within a 7 × 7 cm area. In comparison, *Zhao* and *Rose* used 16 PWAS with resonance frequencies of 350 kHz to detect ice deposits in a circular area with a diameter of 20 cm [28]. In their study, Lamb wave tomographic imaging was applied to detect the ice deposits on a flat plate. With this imaging technique, information on the size and shape of the deposit can be gained in addition to the position. However, tomographic approaches are not well suited for the complex geometry of the bipolar plate investigated in this study, as discussed in Section 3.2.

The environmental and operational conditions (EOCs) during the measurements can influence the guided wave propagation through the bipolar plate. Thus, the difference signal, and ultimately the estimated position of the water drop, depends on the EOCs during the measurements. Ideally, all measurements for the test and training data, as well as the measurement of the baseline signal, should be conducted under the same EOCs. Even though the experiments were conducted in a temperature-controlled lab environment, this criterion was not matched perfectly and small changes in the EOCs still occurred. This can be seen by comparing the seven measurements without any water. The guided wave responses in these measurements were similar but not identical. To investigate the effect of these changes on the localization, each of the seven measurements was used as the baseline measurement to calculate the difference signals. The same signal preprocessing and model training described in Section 4.1 were used for each of the seven baseline signals. The results showed only small effects of the different baseline signals on the localization. In all seven cases, the coefficient of determination $R^{2}$ was 0.92. The estimated *x* and *y* coordinates varied with an average standard deviation of 0.9 mm and 0.7 mm (averaged over all measurements). This is much smaller than the standard deviations of $\delta_{x}$ and $\delta_{y}$ shown in Figure 9a and demonstrates the repeatability of the measurement method.

## 5. Conclusions

In this study, a new measurement method for the localization of liquid water in PEMFCs is proposed. The measurement method is based on ultrasonic guided wave propagation within the bipolar plates of the fuel cell. It is demonstrated in ex situ experiments using a single bipolar plate and a sparse sensor array of four PWAS. By analyzing the guided wave responses and comparing them to Lamb wave theory, it is shown that guided wave propagation is very complex due to the complex geometry of the bipolar plate. Therefore, physical modeling is not a suitable approach to localizing water drops based on the guided wave responses. Instead, data-driven modeling approaches can be applied to extract information on the position of the water drop from the guided wave responses. By applying baseline subtraction, it is shown that the guided wave responses change when a water drop is present on the bipolar plate, leading to an increase in the signal energy of the difference signal. Furthermore, it is shown experimentally that the increase in the signal energy of the difference signal depends on the position of the water drop during the measurement. We have demonstrated the viability of machine learning methods by training a simple linear regression model to localize single water drops on the bipolar plate based on the guided wave responses. The signal energy of the windowed difference signal was used as the model input. The coefficient of determination for the regression model is $R^{2}$ is 0.92. Using the regression model, water drops are located based on the guided wave responses with an empirical standard deviation of 4.2 mm and 3.3 mm in the *x* and *y* directions, respectively.

The measurement method has a high potential for in situ and in operando measurements in a PEMFC cell stack since the small PWAS can be placed directly on the bipolar plates. Compared to other imaging techniques used for the investigation of water deposits in PEMFCs, such as magnetic resonance imaging, X-ray, and neutron imaging [48,49], this approach requires fewer changes to the cell design, can be applied much more easily, and is less costly. Such measurement systems could provide important information on water management in PEMFCs and, therefore, help to increase their durability and performance by optimizing the design and operation. Further research is needed to adapt this measurement principle for in situ measurements. Most importantly, the influence of environmental and operational conditions on the performance of the model has to be evaluated. Temperature changes within the operating PEMFC might require compensation strategies and additional data for the model training. Furthermore, different ML approaches could be applied to localize multiple drops on the bipolar plate and to gain additional information on the size and shape of the drops from the guided wave responses.

In conclusion, our results demonstrate that guided wave responses can be used to localize deposits on geometrically complex structures such as the bipolar plate. In addition to water localization in PEMFCs, this measurement method could be interesting for applications such as fouling localization in plate heat exchangers.

## Data Availability

The data presented in this study are available on request from the corresponding author.

## Abbreviations

The following abbreviations are used in this manuscript:

AISI	American Iron and Steel Institute
DAQ	data acquisition system
EM	electromagnetic
EOCs	environmental and operational conditions
ML	machine learning
MSD	mean squared deviation
PC	personal computer
PEMFC	proton exchange membrane fuel cell
PWAS	piezoelectric wafer active sensors
ROI	region of interest
RSMD	root mean squared deviation
SHM	structural health monitoring
